# Prognostic value of systemic inflammatory markers and development of a nomogram in breast cancer

**DOI:** 10.1371/journal.pone.0200936

**Published:** 2018-07-26

**Authors:** Uiju Cho, Hong Sik Park, So Young Im, Chang Young Yoo, Ji Han Jung, Young Jin Suh, Hyun Joo Choi

**Affiliations:** 1 Department of Hospital Pathology, St. Vincent’s Hospital, College of Medicine, The Catholic University of Korea, Seoul, Republic of Korea; 2 Department of Surgery, St. Vincent’s Hospital, College of Medicine, The Catholic University of Korea, Seoul, Republic of Korea; Sudbury Regional Hospital, CANADA

## Abstract

Systemic inflammatory markers derived from peripheral blood cell, such as the neutrophil-lymphocyte ratio (NLR), derived neutrophil-lymphocyte ratio (dNLR), platelet-lymphocyte ratio (PLR) and lymphocyte-monocyte ratio (LMR), have been demonstrated as prognostic markers in several types of malignancy. Here, we investigated and compared the association between systemic inflammatory markers and survival and developed a prognostic nomogram in breast cancer patients. We reviewed the clinical and pathological records of 661 patients diagnosed with invasive breast carcinoma between 1993 and 2011. The NLR, dNLR, PLR and LMR in the immediate preoperative period were assessed. We analyzed the relationship between these inflammatory markers and clinicopathologic variables, disease-specific survival (DSS), and disease-free survival (DFS) in patients. A nomogram was developed to predict 3- and 5-year DSS for breast cancer. In the univariate analysis, high NLR, dNLR, PLR and low LMR were all significantly associated with poor DSS and DFS. In the multivariate analysis, only the PLR (HR 3.226, 95% CI 1.768–5.885 for DSS and HR 1.824, 95% CI 1.824–6.321 for DFS) was still identified as an independent predictor of outcomes. A subgroup analysis revealed that the PLR was the sole independent marker predicting poor DSS in patients with lymph node metastasis (HR 2.294, 95% CI 1.102–4.777) and with luminal subtype (HR 4.039, 95% CI 1.905–8.562). The proposed nomogram, which includes the PLR, shows good accuracy in predicting DSS with a concordance index of 0.82. PLR is an indicator of systemic inflammation as a part of the host immune response. As an independent prognostic factor, an elevated preoperative PLR is superior to the NLR, dNLR, and LMR in predicting clinical outcomes in patients with breast cancer. Moreover, the nomogram incorporating the PLR could accurately predict individualized survival probability in breast cancer.

## Introduction

Breast cancer is the most common malignancy in women and the 6^th^ leading cause of death in Korean women (http://www.cancer.go.kr/) despite advances in early detection methods and new therapeutic options. It is a heterogeneous disease with variable clinical outcomes and has different genomic subtypes. Substantial effort has been dedicated to subclassifying this heterogeneous disease according to its molecular nature, and treatment plans are now determined according to subtype [1]. Patient prognosis has been greatly improved through these efforts, and as a result, the 5-year relative survival rate of localized breast cancer patients is approximately 100% [2]. However, the 5-year relative survival rate of patients with stage II, II and IV breast cancer drops to 93%, 73% and 22%, respectively [2]. More advanced patient stratification and customized treatment strategy are in need for these patients.

Currently, histopathologic classifications of breast cancer (i.e., tumor grade, stage, histologic type, lymph node status and hormone receptor status) are primarily used to draw correlations with survival; however, they are usually decided postoperatively [3]. Moreover, these classical classifications have little discriminatory prognostic value in patients with advanced breast cancer.

Relatively fewer preoperative biomarkers are recognized as independent prognostic markers. Systemic inflammatory indicators have recently been introduced as reliable and easily performed prognostic markers in several types of cancer [4–6]. Mounting evidence supports the role of inflammation in cancer development, progression, metastasis and treatment resistance [7]. Based on the number of circulating inflammatory cells, some combined indices have been calculated and have been suggested as simple parameters to assess systemic inflammation. One such index is the neutrophil-lymphocyte ratio (NLR), which is correlated with patient prognosis in breast, lung, colorectal, gastric cancer and urothelial carcinoma [8–12]. In previous studies, the NLR was found to be a predictor of worse survival in not only advanced but also in early breast cancer patients [13]. The platelet-lymphocyte ratio (PLR) and lymphocyte-monocyte ratio (LMR) represent other valuable inflammatory indices. The PLR was suggested as a prognostic marker in several types of cancers, including gastric, ovarian, colorectal, pancreatic cancer and cholangiocarcinoma [11,14–19]. In breast cancer, an elevated PLR has been found to adversely impact survival in a few number of studies [6,20]. The value of the LMR as a prognostic marker has been verified in some cancer types, including head and neck cancers, urinary bladder cancer as well as soft tissue sarcomas [21–23]. To date, studies of inflammatory markers have focused more on the NLR, and the roles of the PLR and LMR in breast cancer prognosis are less well known. In addition, no reports have simultaneously compared the prognostic values of the NLR, PLR and LMR in breast cancer.

Therefore, we aimed to verify the relationship between pretreatment inflammatory indices (i.e., the NLR, PLR, and LMR) and the prognoses of patients with breast cancer and to investigate which marker is most useful as a prognostic factor.

## Materials and methods

### Patients

This study evaluated patients who underwent surgery for the treatment of invasive breast cancer at St. Vincent’s Hospital from January 2003 to December 2011. Patients who received neoadjuvant chemotherapy were excluded from the study. All patients were treated with surgical resection and followed standard treatment guidelines as outlined during that timeframe, and patients who received preoperative adjuvant chemotherapy or radiation therapy were excluded from the study. Data regarding patient demographics, clinicopathologic parameters and survival were retrospectively collected from hospital medical records. All samples and medical record data were matched and anonymized before used in this study. Fully anonymized data were accessed by the authors. The use of medical record data and samples for this study was approved by the Institutional Review Board of St. Vincent's Hospital (VC15RISI0190). Informed consent from the participants was waived by IRB of St.Vincent's Hospital (VC15RISI0190).

### Study variables

Preoperative peripheral blood count results (i.e., counts taken during preoperative assessments) obtained within 2 weeks prior to the surgery were extracted from hospital medical records. The NLR was defined as the absolute blood neutrophil count divided by the absolute lymphocyte count, and the derived neutrophil/lymphocyte ratio (dNLR) was defined as the absolute neutrophil count divided by the derived lymphocyte count (absolute leukocyte count—neutrophil count). The PLR was defined as the absolute platelet count divided by the absolute lymphocyte count. The LMR was defined as the absolute lymphocyte count divided by the absolute monocyte count.

Estrogen receptor (ER), progesterone receptor (PR), and HER2 analyses were performed with immunohistochemistry (IHC) and they had been evaluated from whole sections at the time of diagnosis. ER and PR expression levels were scored using the Allred method [24]. An Allred score ≥3 was considered positive. HER2 status was scored as positive if the IHC staining result was 3+ and negative if the staining result was 0 or 1+. Cases with equivocal HER2 status 2+ were subject to fluorescence *in situ* hybridization or silver *in situ* hybridization for confirmation [25]. Each of the intrinsic breast cancer subtypes was classified as follows: luminal type (ER and/or PR positive); HER2 positive type (ER and PR negative, HER2 positive); and triple negative type (ER, PR, and HER2 negative) [1]. Pathologic stages were categorized according to the 7th edition of the TNM classification and stage grouping by the American Joint Committee on Cancer [26].

### Statistical analysis

The χ^2^ test or Fisher’s exact test was used to analyze the correlation between the inflammatory indices and clinicopathologic parameters. Student’s t-test was used to compare different groups of continuous parametric data. Disease-specific survival (DSS) time was measured from the time of initial diagnosis until death due to the breast cancer or until the end of follow-up. Disease-free survival (DFS) time was measured from the time of initial diagnosis until development of new metastatic lesion or disease recurrence. Kaplan-Meier survival graphs and log-rank tests were used to perform univariate survival analysis, and multivariate analysis for the DSS, and DFS were performed using the Cox proportional hazards model. The analyses listed above were performed using SPSS 21.0 (IBM, Armonk, New York, USA). The ideal cutoff values for the NLR, dNLR, PLR and LMR were determined by applying the receiver operating characteristics (ROC) curve analysis. The specificity and sensitivity for the studied outcomes were plotted to generate an ROC curve, and the area under the curve (AUC) for each marker was calculated. A score closest to the point of maximum sensitivity and specificity was selected as the cutoff score leading to the largest group of tumors that were correctly classified as having or not having the DSS. Generation and analysis of the ROC curve were done using MedCalc statistical software package 16.4.3 (MedCalc Software, Ostend, Belgium). Additionally, a nomogram for possible prognostic factors was formulated to provide visualized risk prediction using R software with the survival and rms packages. The performance of the nomogram for predicting survival was evaluated with Harrell’s concordance index (c-index) which is a measure of discrimination. The maximum value of the c-index is 1.0 and it indicates a perfect discrimination. The c-index 0.5 indicates a random chance to correctly discriminate the outcome. Calibration of the nomogram for 5-year DSS was performed by comparing the predicted outcomes with the observed outcomes. Two-tailed P-values <0.05 were considered to be significant.

## Results

### Patient characteristics and inflammatory markers

A total of 661 breast cancer patients were included in the current analysis. Baseline patient characteristics are summarized in Table 1. There were three male patients, and the mean age at the time of diagnosis was 52.7±11.5 years. More than half of the patients (62.2%) were free of lymph node metastasis. At the initial diagnosis, 37.7% of the patients presented with stage I breast cancer, followed by 42.1% with stage II, 17.1% with stage III, and 3.2% with stage IV. The mean leukocyte count was 6.6±2.2×10^9^ cells/L, the mean platelet count was 259.1±61.6×10^9^ cells/L, and the mean lymphocyte count was 2.1±0.8×10^9^ cells/L. Eighteen patients (1.2%) had lymphocytopenia (<1000 cells/μL). The mean values of the inflammatory markers (i.e., the NLR, dNLR, PLR and LMR) were 1.89±1.38, 1.45±0.85, 142.41±192.78 and 6.73±7.13, respectively.

**Table 1:** Baseline characteristics of the patients according to the NLR, dNLR, PLR and LMR.

Variables	Total	NLR		P value	dNLR		P value	PLR		P value	LMR		P value
		≤1.34	>1.34		≤1.34	>1.34		≤185.5	>185.5		≤3.11	>3.11	
		No. of patients			No. of patients			No. of patients			No. of patients		
		229 (34.6%)	432 (65.4%)		354 (53.6%)	307 (46.4%)		579 (87.6%)	82 (12.4%)		41 (6.2%)	620 (93.8%)	
Age													
≤50 years	348 (52.6%)	108 (31.0%)	240 (69.0%)	0.040	172 (49.4%)	176 (50.6%)	0.025	301 (86.5%)	47 (13.5%)	0.366	22 (6.3%)	326 (93.7%)	0.894
>50 years	313 (47.4%)	121 (38.7%)	192 (61.3%)		182 (41.9%)	131 (41.95%)		278 (88.8%)	35 (11.2%)		19 (6.1%)	294 (93.9%)	
Lymphocyte count (10^9^ cells/L)^a^	2.148 (0.821)	2.535 (0.967)	1.942 (0.645)	<0.001	2.402 (0.911)	1.855 (0.581)	<0.001	2.269 (0.791)	1.293 (0.434)	<0.001	1.372 (0.548)	2.201 (0.810)	<0.001
Neutrophil count (10^9^ cells/L)^a^	3.639 (1.691)	2.569 (1.076)	4.206 (1.684)	<0.001	2.823 (1.132)	4.578 (1.741)	<0.001	3.578 (1.548)	4.073 (2.448)	0.013	4.776 (3.115)	3.564 (1.527)	<0.001
Platelet count (10^9^ cells/L)^a^	259.053 (61.584)	248.812 (60.511)	264.481 (61.526)	0.002	251.201 (58.529)	268.107 (63.834)	<0.001	252.242 (55.558)	307.246 (78.762)	<0.001	249.610 (87.644)	259.677 (59.508)	0.311
Monocyte count (10^9^ cells/L)^a^	0.381 (0.175)	0.350 (0.176)	0.398 (0.173)	0.001	0.370 (0.183)	0.394 (0.165)	0.086	0.386 (0.174)	0.348 (0.177)	0.068	0.618 (0.240)	0.365 (0.158)	<0.001
Sex													
Female	658 (99.5%)	228 (34.7%)	430 (65.3%)	0.999^b^	353 (53.6%)	305 (46.4%)	0.600^b^	576 (87.5%)	82 (12.5%)	0.999^b^	41 (6.2%)	616 (93.8%)	0.999^b^
Male	3 (0.5%)	1 (33.3%)	2 (66.7%)		1 (33.3%)	2 (66.7%)		3 (100%)	0		0	3 (100%)	
Operation													
Lumpectomy	362 (54.8%)	126 (34.8%)	236 (65.2%)	0.923	194 (53.6%)	168 (46.4%)	0.984	324 (89.5%)	38 (10.5%)	0.102	15 (4.1%)	347 (95.9%)	0.015
Mastectomy^c^	299 (45.2%)	103 (34.4%)	196 (65.6%)		160 (53.5%)	139 (46.5%)		255 (85.3%)	44 (14.7%)		26 (8.7%)	272 (91.3%)	
Multiplicity													
Solitary tumor	593 (89.7%)	207 (34.9%)	386 (65.1%)	0.675	318 (53.6%)	275 (46.4%)	0.915	518 (87.4%)	75 (12.6%)	0.577	36 (6.1%)	556 (93.9%)	0.600^b^
Multiple tumors	68 (10.3%)	22 (32.4%)	46 (67.6%)		36 (52.9%)	32 (47.1%)		61 (89.7%)	7 (10.3%)		5 (7.4%)	63 (92.6%)	
Histologic grade													
1	183 (27.7%)	62 (33.9%)	121 (66.1%)	0.965	98 (53.6%)	85 (46.4%)	0.533	169 (92.3%)	14 (7.7%)	0.024	8 (4.4%)	175 (95.6%)	0.477
2	265 (40.1%)	93 (35.1%)	172 (64.9%)		148 (55.8%)	117 (44.2%)		222 (83.8%)	43 (16.2%)		18 (6.8%)	246 (93.2%)	
3	213 (32.2%)	74 (34.7%)	139 (65.3%)		108 (50.7%)	105 (49.3%)		188 (88.3%)	25 (11.7%)		15 (7.0%)	198 (93.0%)	
Nuclear grade													
1	79 (12.0%)	26 (32.9%)	53 (67.1%)	0.882	46 (58.2%)	33 (41.8%)	0.564	73 (92.4%)	6 (7.6%)	0.315	4 (5.1%)	75 (94.9%)	0.129
2	341 (51.6%)	121 (35.5%)	220 (64.5%)		184 (54.0%)	157 (46.0%)		294 (86.2%)	47 (13.8%)		16 (4.7%)	324 (95.3%)	
3	241 (36.5%)	82 (34.0%)	159 (66.0%)		124 (51.5%)	307 (46.4%)		212 (88.0%)	29 (12.4%)		21 (8.7%)	220 (93.8%)	
Lymphovascular invasion													
Absent	476 (72.0%)	170 (35.7%)	306 (64.3%)	0.354	262 (55.0%)	214 (45.0%)	0.219	422 (88.7%)	54 (11.3%)	0.184	25 (5.3%)	450 (94.7%)	0.106
Present	185 (28.0%)	59 (31.9%)	126 (68.1%)		92 (49.7%)	93 (50.3%)		157 (84.9%)	28 (12.4%)		16 (8.6%)	169 (91.4%)	
Perineural invasion													
Absent	597 (90.3%)	208 (34.8%)	389 (65.2%)	0.746	323 (54.1%)	274 (45.9%)	0.388	523 (87.6%)	74 (12.4%)	0.981	36 (6.0%)	560 (94.0%)	0.577
Present	64 (9.7%)	21 (32.8%)	43 (67.2%)		43 (65.4%)	33 (51.6%)		56 (87.5%)	8 (12.5%)		5 (7.8%)	59 (92.2%)	
T stage													
T1	335 (50.7%)	115 (34.3%)	220 (65.7%)	0.257	189 (56.4%)	146 (43.6%)	0.172	300 (89.6%)	35 (10.4%)	0.464	17 (5.1%)	318 (94.9%)	<0.001
T2	277 (41.9%)	103 (37.2%)	174 (62.8%)		142 (51.3%)	135 (48.7%)		237 (85.6%)	40 (14.4%)		16 (5.8%)	260 (94.2%)	
T3	44 (6.7%)	10 (2.7%)	34 (77.3%)		19 (43.3%)	25 (56.8%)		38 (86.4%)	6 (13.6%)		5 (11.4%)	39 (88.6%)	
T4	5 (0.8%)	1 (20.0%)	4 (80.0%)		4 (80.0%)	1 (20.0%)		4 (80.0%)	1 (20.0%)		3 (60.0%)	2 (40.0%)	
Lymph node metastasis													
Absent	411 (62.2%)	151 (36.7%)	260 (63.3%)	0.147	231 (56.2%)	180 (43.8%)	0.080	363 (88.3%)	48 (11.7%)	0.467	19 (4.6%)	392 (95.4%)	0.030
Present	250 (37.8%)	78 (31.2%)	172 (68.8%)		123 (49.2%)	127 (50.8%)		216 (86.4%)	34 (13.6%)		22 (8.8%)	227 (91.2%)	
M stage													
M0	640 (96.8%)	228 (35.6%)	412 (64.4%)	0.002^b^	349 (54.5%)	291 (45.5%)	0.007^b^	563 (88.0%)	77 (12.0%)	0.165^b^	36 (5.6%)	604 (94.4%)	0.007^b^
M1	21 (3.2%)	1 (4.8%)	20 (95.2%)		5 (23.8%)	16 (76.2%)		16 (76.2%)	5 (23.8%)		5 (23.8%)	16 (76.2%)	
AJCC stage													
Ⅰ	249 (37.7%)	86 (34.5%)	163 (65.5%)	0.001	141 (56.6%)	108 (43.4%)	0.001	225 (90.4%)	24 (9.6%)	0.135	10 (4.0%)	239 (96.0%)	<0.001
Ⅱ	278 (42.1%)	112 (40.3%)	166 (59.7%)		161 (57.9%)	117 (42.1%)		243 (87.4%)	35 (12.6%)		13 (4.7%)	265 (95.3%)	
Ⅲ	113 (17.1%)	30 (26.5%)	83 (73.5%)		47 (41.6%)	66 (58.4%)		(84.1%)	18 (15.9%)		13 (11.5%)	100 (88.5%)	
Ⅳ	21 (3.2%)	1 (4.8%)	20 (95.2%)		5 (23.8%)	16 (76.2%)		16 (76.2%)	5 (12.4%)		5 (23.8%)	16 (76.2%)	
ER status													
Negative	262 (39.6%)	98 (37.4%)	164 (62.6%)	0.227	147 (56.1%)	115 (43.9%)	0.286	237 (90.5%)	25 (9.5%)	0.070	18 (6.9%)	244 (93.1%)	0.570
Positive	399 (60.4%)	131 (32.8%)	268 (67.2%)		207 (51.9%)	192 (48.1%)		342 (85.7%)	57 (14.3%)		23 (5.8%)	375 (94.2%)	
PR status													
Negative	300 (45.4%)	116 (38.7%)	184 (61.3%)	0.048	176 (58.7%)	124 (41.3%)	0.016	266 (88.7%)	34 (11.3%)	0.446	19 (6.4%)	280 (93.6%)	0.890
Positive	361 (54.6%)	113 (31.3%)	248 (68.7%)		178 (49.3%)	183 (50.7%)		313 (86.7%)	48 (13.3%)		22 (6.1%)	339 (93.9%)	
HER2 status													
Negative	494 (74.7%)	170 (34.4%)	324 (65.6%)	0.830	258 (52.2%)	236 (47.8%)	0.239	432 (87.4%)	62 (12.6%)	0.846	27 (5.5%)	466 (94.5%)	0.179
Positive	167 (25.3%)	59 (35.3%)	108 (64.7%)		96 (57.5%)	71 (42.5%)		147 (88.0%)	20 (12.0%)		14 (8.4%)	153 (91.6%)	
Intrinsic subtype													
Luminal	448 (67.8%)	147 (32.8%)	301 (67.2%)	0.343	233 (52.0%)	215 (48.0%)	0.240	388 (86.6%)	60 (13.4%)	0.536	24 (5.4%)	423 (94.6%)	0.177
HER2 positive	96 (14.5%)	36 (37.5%)	60 (62.5%)		59 (61.5%)	55 (47.0%)		86 (89.6%)	10 (10.4%)		10 (10.4%)	86 (89.6%)	
Triple negative	117 (17.7%)	46 (39.3%)	71 (60.7%)		62 (53.0%)	55 (47.0%)		105 (89.7%)	82 (12.4%)		7 (6.0%)	110 (94.0%)	

NLR, neutrophil-lymphocyte ratio; dNLR, derived neutrophil-lymphocyte ratio; PLR, platelet-lymphocyte ratio; LMR, lymphocyte-monocyte ratio; AJCC, American Joint Committee on Cancer; ER, estrogen receptor; PR, progesterone receptor

^a^The parameters are presented as the mean (standard deviation). Student’s t-test was used for comparisons between the two groups.

^b^Fisher’s exact test was used for comparisons between the two groups.

^c^This variable includes modified radical mastectomy and radical mastectomy.

The ROC curves for DSS were plotted to determine the optimal cutoff values for the NLR, PLR and LMR. As a result, the optimal cutoff value was 1.34 for the NLR (sensitivity 80.65, specificity 36.73, AUC 0.58) and dNLR (sensitivity 59.68, specificity 55.26, AUC 0.57), 185.5 for the PLR (sensitivity 30.65, specificity 89.48, AUC 0.61) and 3.11 for the LMR (sensitivity 19.67, specificity 94.99, AUC 0.54) (S1 Fig). Using these cutoffs, 432 patients (65.4%) had a high NLR, 307 patients (46.4%) had a high dNLR, 82 patients (12.4%) had a high PLR, and 619 patients (93.6%) had a high LMR. Some of the peripheral blood cell counts were significantly different in the high- and low- inflammatory marker groups. A high NLR group had higher platelet count (mean 264.481×10^9^ cells/L vs 248.812×10^9^ cells/L, P = 0.002) and monocyte count (mean 0.398×10^9^ cells/L vs 0.350×10^9^ cells/L, P = 0.001) as well as neutrophil count. Likewise, a high PLR group had higher neutrophil count (mean 4.073 vs 3.578×10^9^ cells/L, P = 0.013) and a high LMR had lower neutrophil count (mean 3.564×10^9^ cells/L vs 4.776×10^9^ cells/L, P<0.001) (Table 1).

The clinicopathologic parameters related to the aggressiveness of the tumor (distant metastasis and advanced AJCC stage) were significantly correlated with high NLR, high dNLR and low LMR but were not correlated with PLR (Table 1).

### Inflammatory markers and prognostic prediction in breast cancer patients

**Fig 1:**
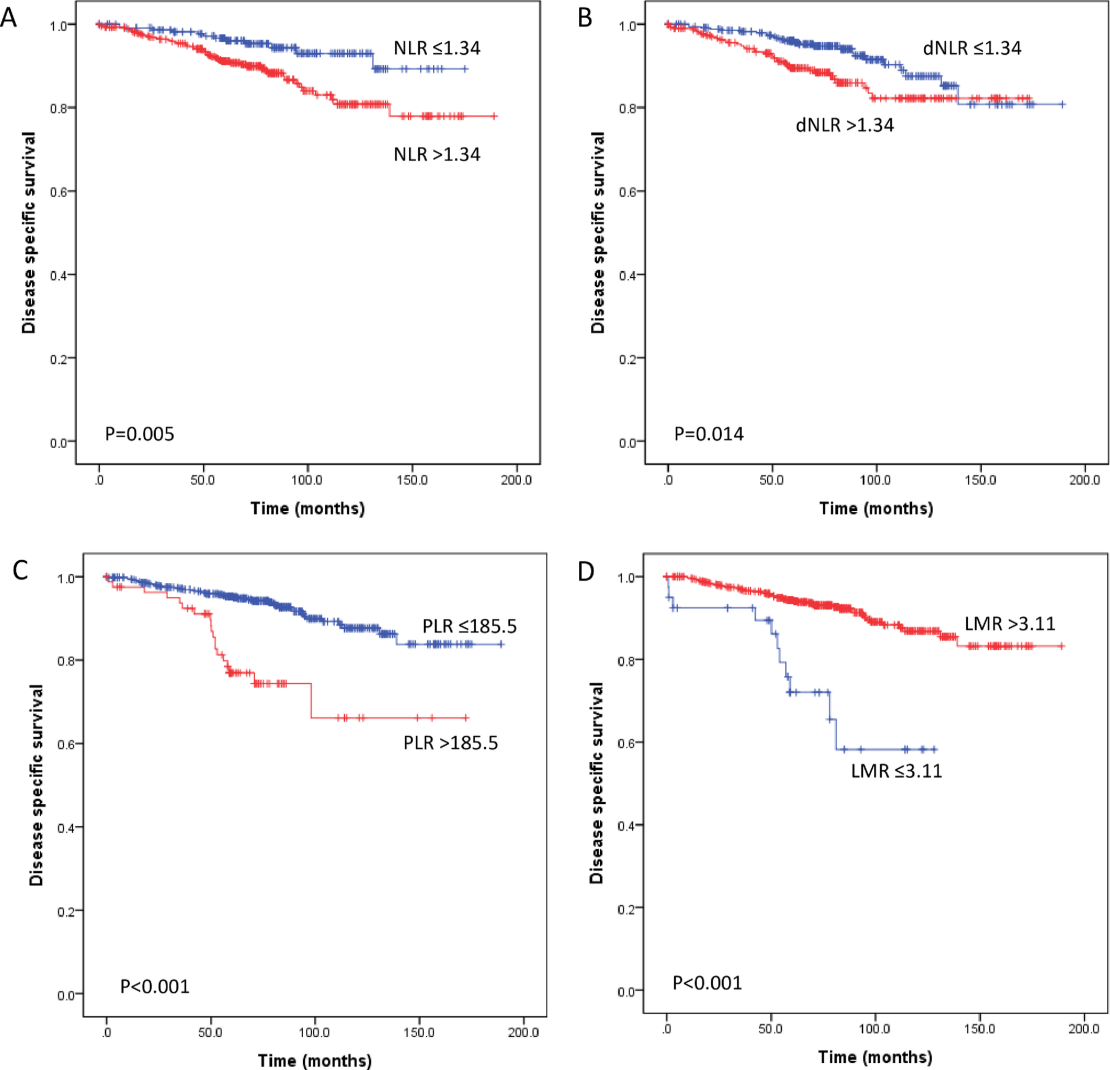
Kaplan-Meier analyses for disease-specific survival of all 661 patients with breast cancer according to the preoperative systemic inflammatory markers. An elevated neutrophil-lymphocyte ratio (NLR) (A), derived neutrophil-lymphocyte ratio (dNLR) (B), and platelet-lymphocyte ratio (PLR) (C) predicted poor disease-specific survival following surgical resection. A low lymphocyte-monocyte ratio (LMR) (D) predicted poor disease-specific survival.

After a median follow-up of 72 months (range, 1–189 months), 110 (16.6%) patients experienced relapse, and 62 (9.4%) patients died of breast cancer. The 5-year DSS and DFS rates were 93% and 83.8%, respectively. The 10-year DSS and DFS rates were 85.2% and 77.8%, respectively. We performed a univariate survival analysis of the inflammatory markers and clinicopathologic parameters. The parameters associated with the DSS of patients with breast cancer included lymphovascular invasion, perineural invasion, T stage, lymph node metastasis, distant metastasis, PR status and all inflammatory markers (i.e., NLR, dNLR, PLR and LMR, all P<0.05) (Table 2) (Fig 1). For DFS, all inflammatory makers (i.e., NLR, dNLR, PLR and LMR), tumor multiplicity, histologic grade, lymphovascular invasion, T stage, lymph node metastasis, distant metastasis and PR expression were associated with prognosis (all P<0.05) (Table 2) (S2 Fig). Prognostic significance of inflammatory markers (i.e., NLR, dNLR, PLR and LMR) and peripheral blood cell counts (i.e., lymphocyte, neutrophil, platelet and monocyte) as a continuous variable was also analyzed by using univariate Cox regression (Table 3). Continuous increase in inflammatory markers (i.e., NLR, dNLR and PLR) were associated with worse DSS and DFS, whereas decrease in lymphocyte count was associated with worse DSS and DFS (all P<0.05). LMR was associated with DSS (P = 0.034) but not with DFS (P = 0.278) as a continuous variable. Neutrophil, platelet and monocyte counts alone were not associated with clinical outcome (all P>0.05).

**Table 2:** Prognostic factors for disease-specific survival and disease-free survival in all 661 patients with breast cancer.

Variables	Disease-specific survival				Disease-free survival			
	Univariate analysis^a^ (P value)	Multivariate analysis^b^			Univariate analysis^a^ (P value)	Multivariate analysis^b^		
		Hazard ratio (95% CI)	Relative risk	P value		Hazard ratio (95% CI)	Relative risk	P value
Nuclear grade	0.43	−	−	−	0.199	−	−	−
ER positive	0.768	−	−	−	0.522	−	−	−
HER2 positive	0.151	−	−	−	0.268	−	−	−
Intrinsic subtype	0.165	−	−	−	0.191	−		−
Multiplicity	0.366	−	−	−	0.033	0.337–2.724	0.959	0.937
Histologic grade (reference 1)	0.244	−	−	−	0.007			0.848
2		−	−	−		0.403–1.790	0.85	0.668
3		−	−	−		0.466–2.157	1.003	0.994
Age (>50 years)	0.059	0.883–2.540	1.497	0.134	0.069	0.850–2.457	1.445	0.174
Lymphovascular invasion	<0.001	0.927–3.303	1.704	0.090	<0.001	0.969–3.474	1.835	0.062
Perineural invasion	0.018	0.620–2.563	1.261	0.522	0.118	−	−	−
T stage (reference 1)	<0.001			0.002	<0.001			0.002
2		0.779–2.741	1.461	0.237		0.788–2.779	1.480	0.223
3		0.984–5.404	2.306	0.054		0.942–5.163	2.205	0.068
4		3.987–78.121	17.649	<0.001		4.069–82.737	18.348	<0.001
Lymph node metastasis	<0.001	0.888–3.351	1.725	0.0107	<0.001	0.903–3.391	1.750	0.097
Distant metastasis (M1)	<0.001	3.571–17.360	7.874	<0.001	<0.001	3.807–18.099	8.301	<0.001
PR positive	0.025	0.326–0.979	0.565	0.042	0.021	0.331–1.082	0.599	0.089
NLR>1.34	0.005	0.532–2.894	1.241	0.681	0.032	0.533–2.905	1.244	0.613
dNLR>1.34	0.014	0.692–2.979	1.436	0.331	0.021	0.665–2.927	1.395	0.378
PLR>185.5	<0.001	1.768–5.885	3.226	<0.001	<0.001	1.824–6.321	1.824	<0.001
LMR≤3.11	<0.001	0.894–4.394	1.497	0.134	0.003	0.908–4.433	2.006	0.085

ER, estrogen receptor; PR, progesterone receptor; NLR, neutrophil-lymphocyte ratio; dNLR, derived neutrophil-lymphocyte ratio; PLR, platelet-lymphocyte ratio; LMR, lymphocyte-monocyte ratio.

^a^Performed using the Kaplan-Meier survival analysis model and log-rank test; values of P<0.10 in the univariate analysis were included in a multivariate analysis.

^b^Performed using Cox proportional hazards model.

**Table 3:** Univariate Cox regression analysis of preoperative inflammatory markers and peripheral blood cell counts.

Variables	Disease-specific survival			Disease-free survival		
	Hazard ratio (95% CI)	Relative risk	P value	Hazard ratio (95% CI)	Relative risk	P value
NLR	1.154–1.391	1.267	<0.001	1.081–1.287	1.180	<0.001
dNLR	1.115–3.079	1.853	0.017	1.057–1.423	1.226	0.007
PLR	1.001–1.002	1.001	<0.001	1.001–1.002	1.001	<0.001
LMR	1.001–1.032	1.016	0.034	0.993–1.026	1.009	0.278
Lymphocyte count (10^9^ cells/L)	0.267–0.619	0.406	<0.001	0.460–0.850	0.625	0.003
Neutrophil count (10^9^ cells/L)	0.849–1.159	0.992	0.917	0.905–1.135	1.013	0.818
Platelet count (10^9^ cells/L)	0.997–1.006	1.001	0.516	0.997–1.003	1.000	0.932
Monocyte count (10^9^ cells/L)	0.031–1.380	0.211	0.104	0.103–1.438	0386	0.156

NLR, neutrophil-lymphocyte ratio; dNLR, derived neutrophil-lymphocyte ratio; PLR, platelet-lymphocyte ratio; LMR, lymphocyte-monocyte ratio

When the patients were grouped by the status of lymphocytopenia (lymphocyte count <1,000/mL), patients with lymphocytopenia had significantly worse prognosis (mean DSS 98.2 months vs 170.1 months, P<0.001; mean DFS 49.2 months vs 136.1 months, P<0.001). Nevertheless, the number of patients with lymphocytopenia was too small (1.2%) to use it as a stratification factor and it was not subjected to succeeding analysis.

Following multivariable adjustment, elevated PLR was identified as an independent predictor of poor DSS (mean survival duration, 133.3 vs 172.2 months; HR = 3.226, P<0.001) and DFS (mean survival duration, 92.1 vs 137.6 months; HR = 1.824, P<0.001) (Table 2).

**Fig 2:**
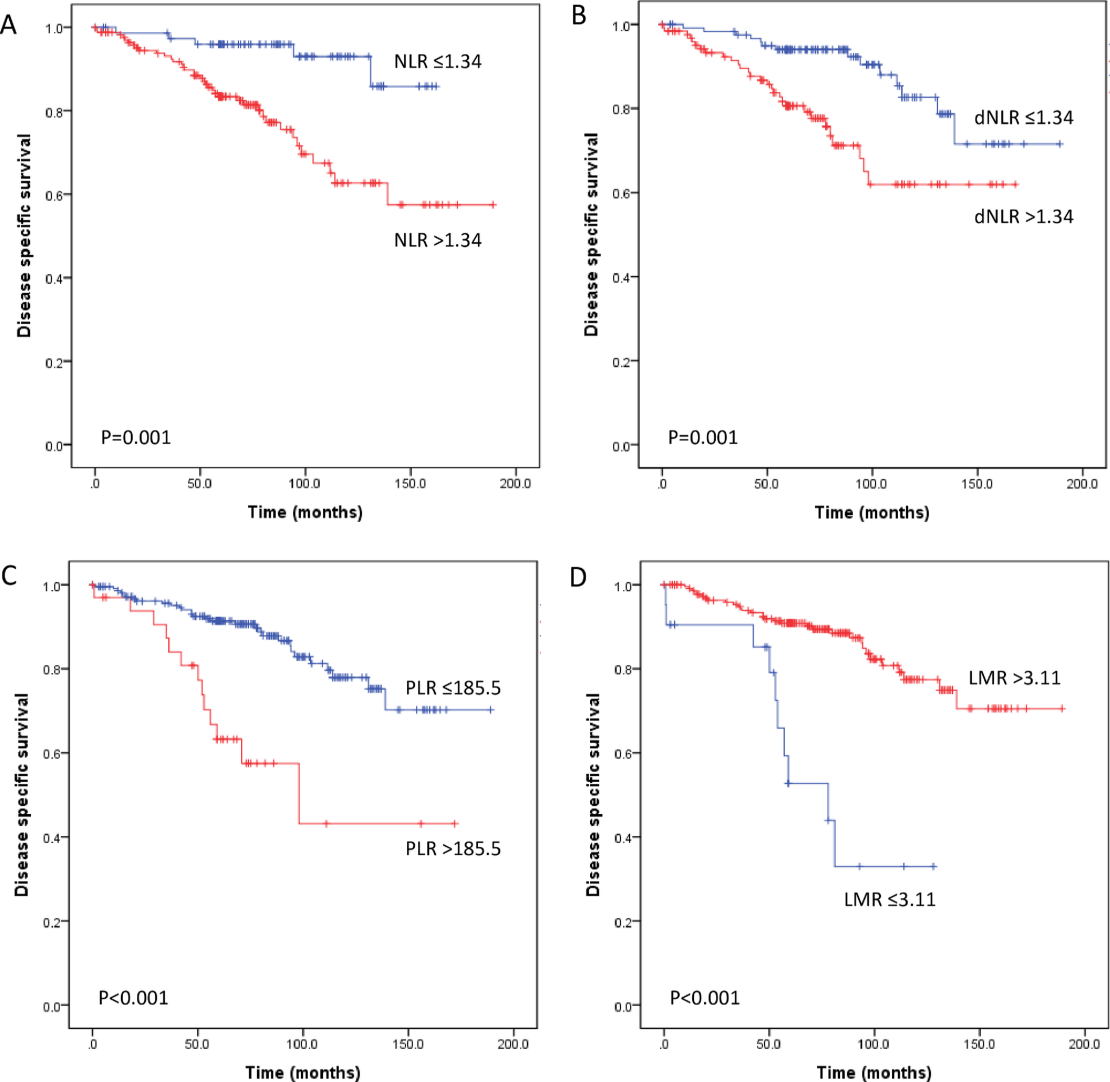
Subgroup analyses of disease-specific survival of 250 patients with lymph node-positive breast cancer according to preoperative systemic inflammatory markers. An elevated neutrophil-lymphocyte ratio (NLR) (A), derived neutrophil-lymphocyte ratio (dNLR) (B), and platelet-lymphocyte ratio (PLR) (C) predicted poor disease-specific survival following surgical resection. A low lymphocyte-monocyte ratio (LMR) (D) predicted poor disease-specific survival. These results aligned with the results of a previous analysis performed with all 661 patients.

We further evaluated the prognostic value of each inflammatory marker in specific subgroups of patients with lymph node metastasis and with different intrinsic subtypes. Of the 250 patients with lymph node metastasis, T stage, distant metastasis, high NLR, dNLR, PLR and a low LMR were associated with worse DSS and DFS in univariate analysis (all P<0.05) (Table 4) (Fig 2 and S3 Fig). Moreover, the PLR and LMR were found to be independent prognostic factors for DSS (HR = 2.294, P = 0.026; HR = 2.916, P = 0.015, respectively). However, the four inflammatory markers were not independently associated with DFS (all P>0.05) (Table 4).

**Table 4:** Prognostic factors for disease-specific survival and disease-free survival in a subgroup of 250 patients with lymph node-positive breast cancer.

Variables	Disease-specific survival				Disease-free survival			
	Univariate analysis^a^ (P value)	Multivariate analysis^b^			Univariate analysis^a^ (p value)	Multivariate analysis^b^		
		Hazard ratio (95% CI)	Relative risk	P value		Hazard ratio (95% CI)	Relative risk	P value
Age (>50 years)	0.593	−	−	−	0.221	−	−	−
Histologic grade	0.875	−	−	−	0.900	−	−	−
Nuclear grade	0.652	−	−	−	0.354	−	−	−
ER positive	0.491	−	−	−	0.542	−	−	−
PR positive	0.381	−	−	−	0.164	−	−	−
Perineural invasion	0.379	−	−	−	0.541	−	−	−
HER2 positive	0.061	0.118–0.888	0.324	0.028	0.206	−	−	−
Lymphovascular invasion	0.089	0.706–2.958	1.445	0.313	0.139	−	−	−
Intrinsic subtype (reference luminal type)	0.276	−	−	−	0.097			
HER2 positive		−	−	−		0.269–1.490	0.633	0.295
Triple negative		−	−	−		0.876–3.213	1.678	0.118
Multiplicity	0.642	−	−	−	0.061	0.100–1.720	0.414	0.225
T stage (reference 1)	<0.001			0.007	<0.001			0.005
2		0.516–2.475	1.131	0.759		0.577–2.005	1.076	0.818
3		0.711–5.071	1.899	0.200		0.873–4.361	1.951	0.103
4		3.594–15.110	21.634	0.001		2.640–41.812	10.506	0.001
Distant metastasis (M1)	<0.001	2.290–15.110	5.883	<0.001	<0.001	2.551–11.623	5.445	<0.001
NLR>1.34	0.001	0.613–5.989	1.915	0.264	0.001	0.807–4.584	1.923	0.140
dNLR>1.34	0.001	0.674–3.685	1.576	0.294	0.007	0.568–2.346	1.155	0.691
PLR>185.5	<0.001	1.102–4.777	2.294	0.026	0.002	0.843–3.220	1.647	0.144
LMR≤3.11	<0.001	1.234–6.889	2.916	0.015	0.001	0.673–3.486	1.531	0.310

ER, estrogen receptor; PR, progesterone receptor; NLR, neutrophil-lymphocyte ratio; dNLR, derived neutrophil-lymphocyte ratio; PLR, platelet-lymphocyte ratio; LMR, lymphocyte-monocyte ratio.

^a^Performed using the Kaplan-Meier survival analysis model and log-rank test; values of P<0.10 in the univariate analysis were included in a multivariate analysis.

^b^Performed using the Cox proportional hazards model.

**Fig 3:**
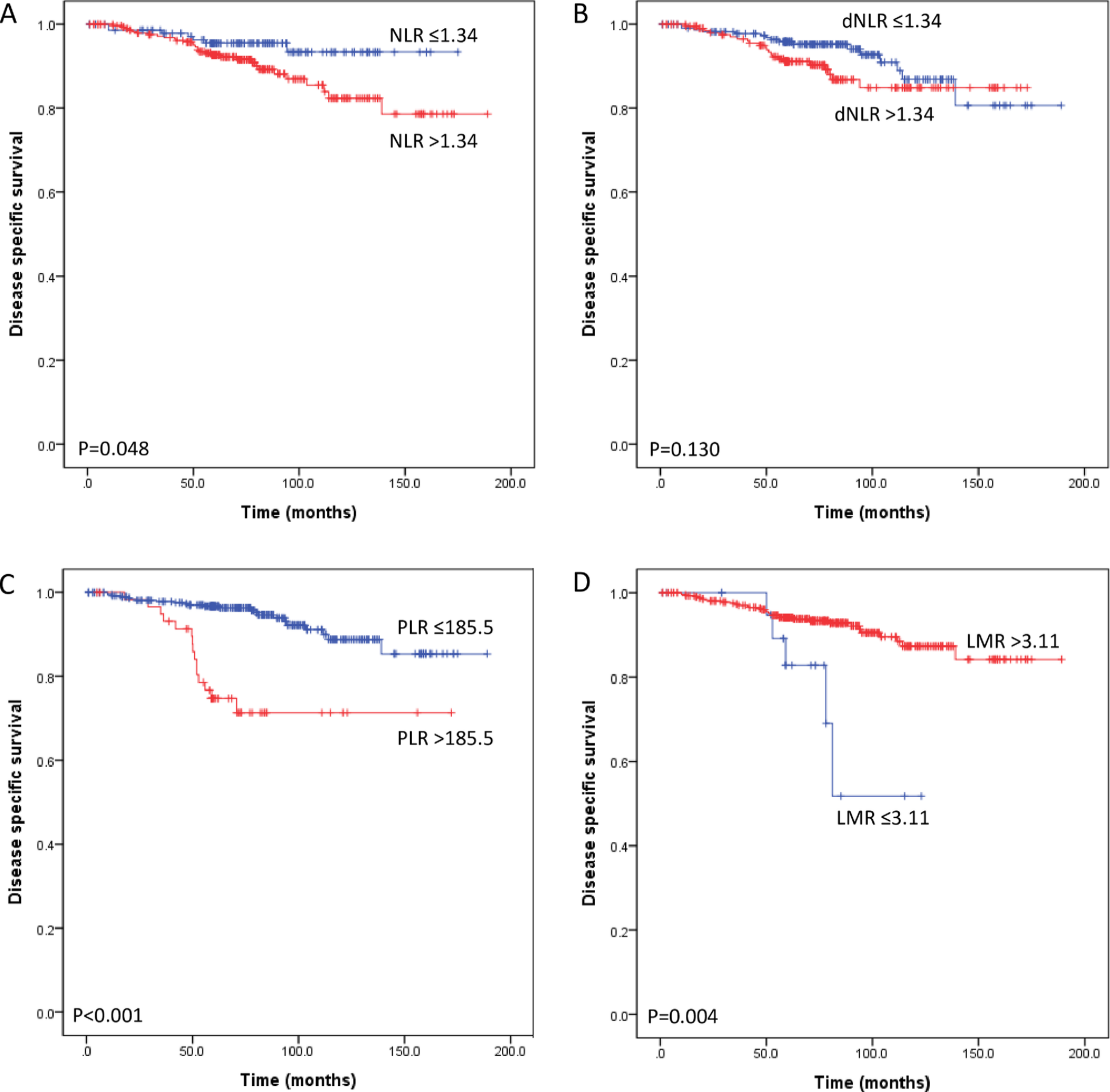
Subgroup analyses of disease-specific survival (DSS) of 448 patients with luminal subtype breast cancer according to preoperative systemic inflammatory markers. An elevated neutrophil-lymphocyte ratio (NLR) (A) predicted poor DSS following surgical resection. Derived NLR (B) did not make significant difference DSS between low and high dNLR groups. A high platelet-lymphocyte ratio (PLR) (C) and a low lymphocyte-monocyte ratio (LMR) (D) predicted poor DSS.

In three intrinsic subtype groups, luminal subtype group had PLR as an independent prognostic factor. Of the 448 patients with luminal subtype breast cancer, a high NLR and PLR were associated with worse DSS (P = 0.048 and P<0.001, respectively) (Fig 3) and DFS (P = 0.044 and P<0.001, respectively) (S4 Fig) but only PLR remained significant after adjusting for other clinicopathologic markers (P<0.001) (Table 5). In the HER2 positive subtype, Kaplan-Meier survival curves for DSS and DFS was significantly different according to LMR (P<0.001 and P = 0.003, respectively) but LMR was not significant prognostic factor in multivariate analyses (all P>0.05). Other inflammatory markers (i.e., NLR, dNLR and PLR) were not associated with clinical outcome in HER2 positive subtype and triple negative subtype (all P>0.05).

**Table 5:** Prognostic factors for disease-specific survival and disease-free survival in a subgroup of 448 patients with luminal type breast cancer.

Variables	Disease-specific survival				Disease-free survival			
	Univariate analysis^a^ (P value)	Multivariate analysis^b^			Univariate analysis^a^ (P value)	Multivariate analysis^b^		
		Hazard ratio (95% CI)	Relative risk	P value		Hazard ratio (95% CI)	Relative risk	P value
Nuclear grade	0.616	−	−	−	0.264	−	−	−
HER2 positive	0.156	−	−	−	0.248	−	−	−
Multiplicity	0.578	−	−	−	0.053	0.313–3.782	1.088	0.895
Histologic grade (reference 1)	0.169	−	−	−	0.009			0.331
2		−	−	−		0.309–1.811	0.747	0.519
3		−	−	−		0.535–3.759	1.418	0.483
Age (>50 years)	0.015	1.028–4.019	2.032	0.042	0.045	0.961–3.830	1.919	0.065
Perineural invasion	0.118	−	−	−	0.381	−	−	−
Lymphovascular invasion	<0.001	0.663–3.382	1.497	0.331	0.001	0.604–3.179	1.386	0.441
T stage (reference 1)	<0.001			0.003	<0.001			0.003
2		0.544–2.684	1.208	0.643		0.550–2.783	1.237	0.607
3		0.991–7.111	2.655	0.052		1.087–8.256	2.996	0.034
4		5.018–482.445	49.203	0.001		4.781–488.357	48.320	0.607
Lymph node metastasis	<0.001	1.257–7.667	3.104	0.014	0.001	1.244–7.624	3.080	0.015
Distant metastasis (M1)	<0.001	1.509–16.715	5.021	0.009	<0.001	1.603–17.433	5.286	0.006
NLR>1.34	0.048	0.591–3.776	1.493	0.397	0.044	0.550–3.525	1.392	0.485
dNLR>1.34	0.130	−	−	−	0.102	−	−	−
PLR.>185.5	<0.001	1.905–8.562	4.039	<0.001	<0.001	2.108–10.497	4.704	<0.001
LMR≤3.11	0.004	0.165–1.308	0.465	0.147	0.091	0.177–1.574	0.527	0.251

NLR, neutrophil-lymphocyte ratio; dNLR, derived neutrophil-lymphocyte ratio; PLR, platelet-lymphocyte ratio; LMR, lymphocyte-monocyte ratio.

^a^Performed using the Kaplan-Meier survival analysis model and log-rank test; values of P<0.10 in the univariate analysis were included in a multivariate analysis.

^b^Performed using the Cox proportional hazards model.

### Nomogram for the prediction of disease-specific survival

**Fig 4:**
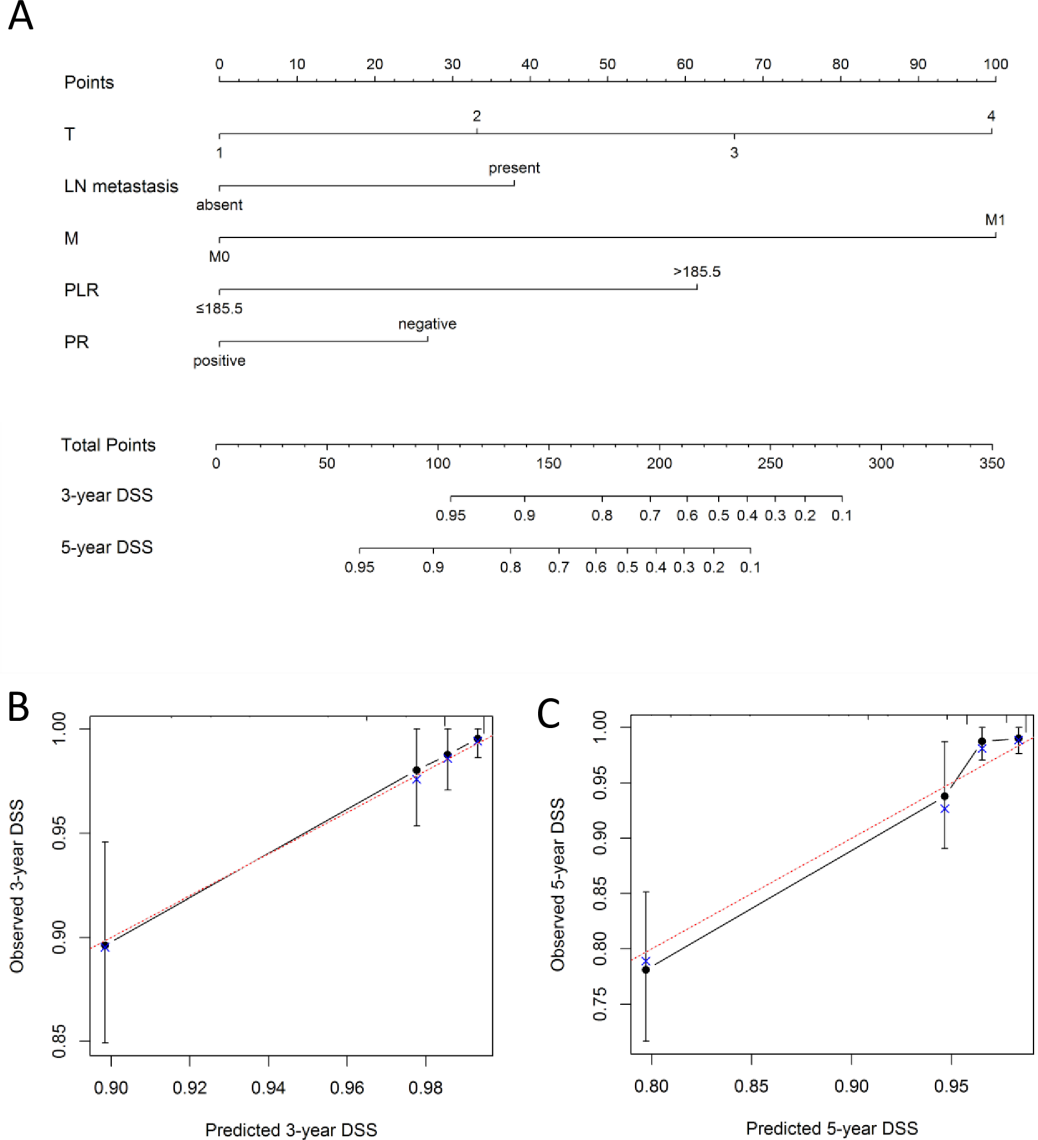
(A) A nomogram for 3- and 5-year disease-specific survival (DSS) for breast cancer patients, including data derived from 661 patients and 62 mortality events. Nomograms can be interpreted by adding up the points assigned to each variable, as indicated at the top of the point scale. The total point projected on the bottom scale represents the probability of 3- or 5-year DSS. Calibration curves for 3-year DSS (B) and 5-year DSS (C) using nomograms with clinicopathological characteristics and pretreatment PLR are shown. The x-axis is nomogram-predicted probability of survival and y-axis is actual survival. The bootstrapping method was used for the internal validation of the nomogram. The red line indicates perfect calibration. T, T stage; LN, lymph node; M, M stage; PLR, platelet-lymphocyte ratio; PR, progesterone receptor, DSS, disease-specific survival.

To predict the disease-specific survival outcomes of breast cancer patients, a prognostic nomogram was established through Cox regression model analysis according to all significant independent indicators of DSS (i.e., T stage, M stage, lymph node metastasis, PR expression and PLR). Each factor in the nomogram was assigned a weighted number of points, and the sum of points for each patient was in accordance with a specific predicted 3- and 5-year DSS. For internal validation, the bootstrapped calibration plot of the nomogram predicting 3- and 5-year DSS performed well with the ideal model (Fig 4). The C-index of the multivariate prognostic model based on T stage, M stage, lymph node metastasis, PR expression was 0.77 (95% CI 0.71–0.83), but it was improved to 0.82 (95% CI 0.77–0.88) when PLR was included in the model.

## Discussion

In the present study, we validated the indices of systemic inflammation (i.e., NLR, dNLR, PLR and LMR) as a prognostic marker of breast cancer. All four markers were significantly associated with DSS and DFS in the patients with breast cancer. However, a high PLR was the only inflammatory maker that was independently associated with worse DSS and DFS in the all-patient group. In patients with lymph node metastasis and with luminal subtype breast cancer, high PLR had a significant prognostic value in DSS according to the multivariate analysis. Peripheral blood cell counts (e.g., neutrophil, platelet and monocyte count) alone were not associated with survival but the inflammation marker that are produced by combination of these cell counts were all significantly associated with survival. To the best of our knowledge, this study is the first to simultaneously compare inflammatory markers (i.e., NLR, dNLR, PLR and LMR) of breast cancer and to further establish a nomogram containing PLR to predict patient survival.

In recent years, the importance of patient-related factors has been recognized, particularly the host response to systemic inflammation in determining disease outcomes in cancer patients [27]. It is now clear that the outcomes of cancer patients are not solely determined by tumor characteristics. Investigators have shown that the pretreatment index or systemic inflammation score can predict survival in patients with several types of cancers [5,28–30]. In particular, white cell counts (i.e., neutrophils, lymphocytes, platelets and monocytes) and their combinations (e.g., NLR, PLR and LMR) have been highlighted because hematological tests are routinely performed for cancer patients in clinical practice, and biologically, the activation of systemic inflammation is associated with changes in circulating white blood cells, such as the occurrence of neutrophilia with associated lymphocytopenia [31]. These systemic inflammation markers are thought to represent an activation of the innate immune/inflammation cascade in these patients [32]. As with neutrophils, many of the cytokines that stimulate neutrophil production from bone marrow are elevated due to cancer. Cancer cells themselves aberrantly produce these molecules, including IL-1β, and surrounding stromal cell and immune cells also significantly contribute to elevated expression levels [33]. Altered inflammation promotes tumor initiation and growth and can also play a pro-metastatic role [33,34].

Additionally, recently discovered evidence has revealed the role of platelets in inflammatory diseases and malignant tumors as well as in hemostasis. Platelets contribute to sustaining proliferative signals, and cancer cells produce platelet-derived growth factors in large quantities. These growth factors are found to promote tumor progression [35]. Elevated platelet counts in peripheral blood have been found to be associated with a worse prognosis in patients with lung cancer [36]. In a meta-analysis, a high PLR was associated with worse overall survival (OS) for several solid tumors (e.g., colorectal, gastroesophageal, ovarian, pancreatic and hepatocellular carcinoma) but not for breast cancer [5]. A different meta-analysis study focused on the prognostic value of PLR on breast cancer and analyzed 7 published articles. The combined results demonstrated that PLR was a valid prognostic biomarker for poor OS and DFS without significant publication bias [37]. However, the number of previous studies included in meta-analyses is too small and consisted of heterogenous patient characteristics. In our study, a high PLR was not correlated with tumor stage or lymph node metastasis. However, a high PLR (>185.5) independently predicted poor DSS and DFS in breast cancer patients (HR = 3.226, P<0.001 and HR = 1.824, P<0.001, respectively). Furthermore, a high PLR demonstrated a strong prognostic value for DSS in patients with metastatic lymph nodes (HR 2.294, P = 0.026) and with luminal subtype (HR 4.039, P<0.001). These data validate the value of PLR as a prognostic biomarker in later-stage breast cancer and align with the results of previous breast cancer studies [20,38].

Prognostic value of PLR in each molecular subtype is not well known. Our data demonstrated PLR as a significant prognostic marker in the luminal subtype. In a meta-analysis of 12 studies, there was a significant difference in the incidence of high levels of PLR between HER2 statuses, but not between ER or PR receptor statuses [39]. High PLR tended to have a lower effect of DFS on ER-/PR- and HER2+ breast cancer but statistical significance was not reached. Moreover, subgroup analysis for OS on the basis of the receptor status was not performed due to the small number of studies. As for NLR, previous studies suggest strong prognostic effect in triple negative breast cancer [13,38] but comprehensive study about the inflammatory markers and molecular subtypes, especially PLR, are necessary to more fully understand their biology.

Nevertheless, few comparative analyses of the potential prognostic value of inflammatory markers in breast cancer have been performed. The PLR was a superior prognostic marker compared to the NLR in colorectal cancer, urinary bladder cancer, pancreatic ductal adenocarcinoma and thyroid medullary carcinoma [17,23,40,41]; however, opposite results have been reported in other studies [15,42–44]. In breast cancer, the prognostic values of NLR vs PLR and NLR vs LMR have been investigated by only a few previous studies [6,38,45–47]. One study reported that an NLR>2.57 is an independent prognostic marker for OS in breast cancer, whereas a PLR>107.64 is not [45]. Also, NLR was found to be a superior predictor of long-term mortality since it continued to be a significant marker regardless of the lymphocyte count status of the patients [46]. In two other studies, the NLR and PLR were associated with OS after multivariate adjustments [6,47] A study by Wariss *et al*. showed that the PLR predicts the risk of death in a statistically significant manner, along with the NLR, and the PLR has a higher adjusted HR than that of the NLR (1.66 vs 1.82, respectively) [47]. In a Glasgow inflammation outcome study, investigators compared several inflammation-based prognostic scores (e.g., modified Glasgow prognostic score, NLR, PLR, prognostic index and PNI) in a large cohort of cancer patients. Elevation of these markers was independently predictive of DSS in the breast cancer and in other tumor sites. The NLR had greater AUC than PLR (0.640 vs 0.638, respectively) in this study but the AUC was greatest for modified Glasgow prognostic score (0.712) [48]. Regarding the NLR and LMR, the prognostic values of these markers were co-analyzed in 1570 operable breast cancer patients, and they were both significantly associated with DFS in a univariate analysis [38]. However, only the low NLR was a significant independent predictor of superior OS and DFS in all patients and in triple negative breast cancer patients [38]. In our study, all the inflammatory markers did not have prognostic significance in triple negative subtype. Our results regarding the prognostic value of the PLR also differed from those of previous studies. The PLR was not only a significant independent prognostic marker but also more superior marker than the NLR in the all patient group, lymph node-positive and luminal subtype patient group. This finding may be partly because of differences in the study design and ethnicities.

No other studies have simultaneously adjusted the NLR, PLR and LMR in a cohort of breast cancer patients except for the present one, and most other studies have empirically selected the cutoff values of inflammatory markers. Differences in the selected cutoff values make a direct comparison of the study results difficult. Unlike previous studies, we determined the ideal cutoff values of the NLR, PLR and LMR based on the ROC curve and were able to use the AUC to compare the predictive power of the inflammatory markers (0.58, 0.61 and 0.54, respectively). Still, we have not analyzed inflammation-based prognostic scores, especially the ones that use C-reactive protein such as modified Glasgow prognostic score, to draw a conclusion that PLR is the best single prognostic parameter in breast cancer. It would be of interest to investigate and appraise for the most efficient choice of inflammation marker that should be included in the routine assessment of breast cancer patients.

Our data also support the notion that relative lymphocytopenia is associated with poor outcomes in cancer patients [49,50]. In a previous breast cancer study, lymphocytopenia before treatment had significant correlation with OS in patients with metastatic breast cancer (P<0.0001) in a multivariate analysis [50]. Lymphocytopenia was also related to tumor burden, metastatic sites, paraneoplastic inflammatory syndrome and host characteristics [50]. In our study, patient groups with inferior outcome (high NLR, dNLR, PLR and low LMR) in the univariate analysis had significantly lower numbers of peripheral blood lymphocytes (Table 1). However, the number of patients with significant lymphocytopenia was too small (1.2%) to use it as a stratification factor. Anyhow, a lymphocytopenia was significantly associated with worse DSS (P<0.001) (data not shown in the results). The mechanisms of pretreatment lymphocytopenia and the association between lymphocytopenia and prognosis in cancer patients remain elusive. Lymphocytopenia may be due to the apoptosis of lymphocytes drawn by cancer cells using the Fas/Fas-ligand pathway or through an alteration of the hemostasis of lymphocytes [51,52].

The performance of a nomogram must be evaluated through calibration and discrimination. In this study, internal validation showed good discrimination power (C-index, 0.82) when PLR was included in the nomogram. Our nomogram was well calibrated to predict DSS.

Our study has some limitations as a retrospective, single hospital and relatively small-sized sample. In addition, leukocyte counts can be influenced by other medical conditions or factors, such as infection, medication, malnutrition, severe stress and non-malignant inflammatory diseases. Our data should be interpreted with caution because our study design may not exclude these factors. Moreover, it lacks an external validation cohort, which could further confirm its robustness beyond the present data. Larger prospective studies are required to confirm these preliminary results and investigation of the relationship between peripheral inflammatory marker and tumor-infiltrating lymphocyte could further expand our understanding about the breast cancer biology.

In conclusion, we have demonstrated that the PLR is an independent prognostic marker for survival in all breast cancer patients, lymph node-positive and luminal type breast cancer patients. Furthermore, the nomogram incorporating PLR accurately predicted individualized survival probability in breast cancer. This practical model could support clinicians and patients in clinical decision-making and treatment optimization.

## Supporting information

S1 Fig Receiver operating characteristic (ROC) curves for disease specific survival. (DOCX)

S2 Fig Kaplan-Meier analyses for disease-free survival of all 661 patients with breast cancer, according to the preoperative systemic inflammatory marker. (DOCX)

S3 Fig Subgroup analyses of the disease-free survival of 250 patients with lymph node-positive breast cancer according to preoperative systemic inflammatory markers. (DOCX)

S4 Fig Subgroup analyses of the disease-free survival of 448 patients with luminal type breast cancer according to preoperative systemic inflammatory markers. (DOCX)

S1 Table Raw data. (XLSX)

## References

[1] Goldhirsch A, Wood WC, Coates AS, Gelber RD, Thurlimann B, Senn HJ. Strategies for subtypes—dealing with the diversity of breast cancer: highlights of the St. Gallen International Expert Consensus on the Primary Therapy of Early Breast Cancer 2011. Ann Oncol. 2011; 22(8):1736–47. 10.1093/annonc/mdr304 21709140 PMC3144634

[2] Howlader N NA. SEER Cancer Statistics Review, 1975–2014, National Cancer Institute. Bethesda, MD. 2016 Nov SEER data submission [cited April 2017]. In: SEER web site. Available from https://seer.cancer.gov/csr/1975_2014/.

[3] Elston CW, Ellis IO. Pathological prognostic factors in breast cancer. I. The value of histological grade in breast cancer: experience from a large study with long-term follow-up. Histopathology. 1991; 19(5):403–10. 1757079 10.1111/j.1365-2559.1991.tb00229.x

[4] Teng J-J, Zhang J, Zhang T-Y, Zhang S, Li B-S. Prognostic value of peripheral blood lymphocyte-to-monocyte ratio in patients with solid tumors: a meta-analysis. OncoTargets and therapy. 2016; 9:37–47. PMID: PMC4694666 10.2147/OTT.S94458 26730202 PMC4694666

[5] Templeton AJ, Ace O, McNamara MG, Al-Mubarak M, Vera-Badillo FE, Hermanns T. Prognostic role of platelet to lymphocyte ratio in solid tumors: a systematic review and meta-analysis. Cancer Epidemiol Biomarkers Prev. 2014; 23 10.1158/1055-9965.epi-14-0146 24793958

[6] Koh CH, Bhoo-Pathy N, Ng KL, Jabir RS, Tan GH, See MH, et al Utility of pre-treatment neutrophil-lymphocyte ratio and platelet-lymphocyte ratio as prognostic factors in breast cancer. Br J Cancer. 2015; 113(1):150–8. 10.1038/bjc.2015.183 26022929 PMC4647546

[7] Crusz SM, Balkwill FR. Inflammation and cancer: advances and new agents. Nat Rev Clin Oncol. 2015; 12(10):584–96. 10.1038/nrclinonc.2015.105 26122183

[8] Azab B, Bhatt VR, Phookan J, Murukutla S, Kohn N, Terjanian T, et al Usefulness of the neutrophil-to-lymphocyte ratio in predicting short- and long-term mortality in breast cancer patients. Ann Surg Oncol. 2012; 19(1):217–24. 10.1245/s10434-011-1814-0 21638095

[9] Teramukai S, Kitano T, Kishida Y, Kawahara M, Kubota K, Komuta K. Pretreatment neutrophil count as an independent prognostic factor in advanced non-small-cell lung cancer: an analysis of Japan Multinational Trial Organisation LC00-03. Eur J Cancer. 2009; 45 10.1016/j.ejca.2009.01.023 19231158

[10] Kishi Y, Kopetz S, Chun YS, Palavecino M, Abdalla EK, Vauthey JN. Blood neutrophil-to-lymphocyte ratio predicts survival in patients with colorectal liver metastases treated with systemic chemotherapy. Ann Surg Oncol. 2009; 16 10.1245/s10434-008-0267-6 19130139

[11] Lee S, Oh SY, Kim SH, Lee JH, Kim MC, Kim KH, et al Prognostic significance of neutrophil lymphocyte ratio and platelet lymphocyte ratio in advanced gastric cancer patients treated with FOLFOX chemotherapy. BMC Cancer. 2013; 13:350 10.1186/1471-2407-13-350 23876227 PMC3729814

[12] Hermanns T, Bhindi B, Wei Y, Yu J, Noon AP, Richard PO, et al Pre-treatment neutrophil-to-lymphocyte ratio as predictor of adverse outcomes in patients undergoing radical cystectomy for urothelial carcinoma of the bladder. Br J Cancer. 2014; 111(3):444–51. 10.1038/bjc.2014.305 24918819 PMC4119979

[13] Pistelli M, De Lisa M, Ballatore Z, Caramanti M, Pagliacci A, Battelli N, et al Pre-treatment neutrophil to lymphocyte ratio may be a useful tool in predicting survival in early triple negative breast cancer patients. BMC Cancer. 2015; 15:195 10.1186/s12885-015-1204-2 25884918 PMC4428113

[14] Asher V, Lee J, Innamaa A, Bali A. Preoperative platelet lymphocyte ratio as an independent prognostic marker in ovarian cancer. Clin Transl Oncol. 2011; 13 10.1007/s12094-011-0687-9 21775277

[15] Zou Z-Y, Liu H-L, Ning N, Li S-Y, Du X-H, Li R. Clinical significance of pre-operative neutrophil lymphocyte ratio and platelet lymphocyte ratio as prognostic factors for patients with colorectal cancer. Oncology Letters. 2016; 11(3):2241–8. PMID: PMC4774601 10.3892/ol.2016.4216 26998156 PMC4774601

[16] Dominguez I, Crippa S, Thayer SP, Hung YP, Ferrone CR, Warshaw AL. Preoperative platelet count and survival prognosis in resected pancreatic ductal adenocarcinoma. World J Surg. 2008; 32 10.1007/s00268-007-9423-6 PMC3806089 18224462

[17] Smith RA, Bosonnet L, Raraty M, Sutton R, Neoptolemos JP, Campbell F, et al Preoperative platelet-lymphocyte ratio is an independent significant prognostic marker in resected pancreatic ductal adenocarcinoma. The American Journal of Surgery. 2009; 197(4):466–72. 10.1016/j.amjsurg.2007.12.057 18639229

[18] Dolan RD, McSorley ST, Horgan PG, Laird B, McMillan DC. The role of the systemic inflammatory response in predicting outcomes in patients with advanced inoperable cancer: Systematic review and meta-analysis. Crit Rev Oncol Hematol. 2017; 116:134–46. 10.1016/j.critrevonc.2017.06.002 28693795

[19] Zhou X, Du Y, Huang Z, Xu J, Qiu T, Wang J, et al Prognostic value of PLR in various cancers: a meta-analysis. PLoS One. 2014; 9(6):e101119 10.1371/journal.pone.0101119 24968121 PMC4072728

[20] Krenn-Pilko S, Langsenlehner U, Thurner EM, Stojakovic T, Pichler M, Gerger A, et al The elevated preoperative platelet-to-lymphocyte ratio predicts poor prognosis in breast cancer patients. British Journal of Cancer. 2014; 110(10):2524–30. PMID: PMC4021515 10.1038/bjc.2014.163 24675383 PMC4021515

[21] Szkandera J, Gerger A, Liegl-Atzwanger B, Absenger G, Stotz M, Friesenbichler J. The lymphocyte/monocyte ratio predicts poor clinical outcome and improves the predictive accuracy in patients with soft tissue sarcomas. Int J Cancer. 2014; 135 10.1002/ijc.28677 24347236

[22] Kano S, Homma A, Hatakeyama H, Mizumachi T, Sakashita T, Kakizaki T, et al Pretreatment lymphocyte-to-monocyte ratio as an independent prognostic factor for head and neck cancer. Head Neck. 2016 10.1002/hed.24576 27617428

[23] Zhang GM, Zhu Y, Luo L, Wan FN, Zhu YP, Sun LJ, et al Preoperative lymphocyte-monocyte and platelet-lymphocyte ratios as predictors of overall survival in patients with bladder cancer undergoing radical cystectomy. Tumour Biol. 2015; 36(11):8537–43. 10.1007/s13277-015-3613-x 26032095

[24] Allred DC, Harvey JM, Berardo M, Clark GM. Prognostic and predictive factors in breast cancer by immunohistochemical analysis. Mod Pathol. 1998; 11(2):155–68. 9504686

[25] Papouchado BG, Myles J, Lloyd RV, Stoler M, Oliveira AM, Downs-Kelly E, et al Silver in situ hybridization (SISH) for determination of HER2 gene status in breast carcinoma: comparison with FISH and assessment of interobserver reproducibility. Am J Surg Pathol. 2010; 34(6):767–76. 10.1097/PAS.0b013e3181d96231 20421783

[26] Edge S. AJCC Cancer Staging Manual 7th ed New York: Springer; 2010.

[27] Hanahan D, Weinberg RA. Hallmarks of cancer: the next generation. Cell. 2011; 144(5):646–74. 10.1016/j.cell.2011.02.013 21376230

[28] Szkandera J, Absenger G, Liegl-Atzwanger B, Pichler M, Stotz M, Samonigg H. Elevated preoperative neutrophil/lymphocyte ratio is associated with poor prognosis in soft-tissue sarcoma patients. Br J Cancer. 2013; 108 10.1038/bjc.2013.135 PMC3668478 23558897

[29] Allin KH, Nordestgaard BG, Flyger H, Bojesen SE. Elevated pre-treatment levels of plasma C-reactive protein are associated with poor prognosis after breast cancer: a cohort study. Breast Cancer Res. 2011; 13 10.1186/bcr2891 PMC3218944 21639875

[30] Guthrie GJ, Charles KA, Roxburgh CS, Horgan PG, McMillan DC, Clarke SJ. The systemic inflammation-based neutrophil-lymphocyte ratio: experience in patients with cancer. Crit Rev Oncol Hematol. 2013; 88 10.1016/j.critrevonc.2013.03.010 23602134

[31] Gabay C, Kushner I. Acute-phase proteins and other systemic responses to inflammation. N Engl J Med. 1999; 340(6):448–54. 10.1056/NEJM199902113400607 9971870

[32] Roxburgh CS, McMillan DC. Cancer and systemic inflammation: treat the tumour and treat the host. Br J Cancer. 2014; 110(6):1409–12. 10.1038/bjc.2014.90 24548867 PMC3960633

[33] Coffelt SB, Wellenstein MD, de Visser KE. Neutrophils in cancer: neutral no more. Nat Rev Cancer. 2016; 16(7):431–46. 10.1038/nrc.2016.52 27282249

[34] Franco AT, Corken A, Ware J. Platelets at the interface of thrombosis, inflammation, and cancer. Blood. 2015; 126(5):582–8. 10.1182/blood-2014-08-531582 26109205 PMC4520875

[35] Ishii Y, Hamashima T, Yamamoto S, Sasahara M. Pathogenetic significance and possibility as a therapeutic target of platelet derived growth factor. Pathol Int. 2017; 67(5):235–46. 10.1111/pin.12530 28393435

[36] Radziwon-Balicka A, Medina C, O'Driscoll L, Treumann A, Bazou D, Inkielewicz-Stepniak I. Platelets increase survival of adenocarcinoma cells challenged with anticancer drugs: mechanisms and implications for chemoresistance. Br J Pharmacol. 2012; 167 10.1111/j.1476-5381.2012.01991.x PMC3575779 22506717

[37] Zhu Y, Si W, Sun Q, Qin B, Zhao W, Yang J. Platelet-lymphocyte ratio acts as an indicator of poor prognosis in patients with breast cancer. Oncotarget. 2017; 8(1):1023–30. PMID: PMC5352031 10.18632/oncotarget.13714 27906679 PMC5352031

[38] Jia W, Wu J, Jia H, Yang Y, Zhang X, Chen K, et al The Peripheral Blood Neutrophil-To-Lymphocyte Ratio Is Superior to the Lymphocyte-To-Monocyte Ratio for Predicting the Long-Term Survival of Triple-Negative Breast Cancer Patients. PLoS One. 2015; 10(11):e0143061 10.1371/journal.pone.0143061 26580962 PMC4666347

[39] Zhang M, Huang XZ, Song YX, Gao P, Sun JX, Wang ZN. High Platelet-to-Lymphocyte Ratio Predicts Poor Prognosis and Clinicopathological Characteristics in Patients with Breast Cancer: A Meta-Analysis. Biomed Res Int. 2017; 2017:9503025 10.1155/2017/9503025 29082257 PMC5610825

[40] Kwon HC, Kim SH, Oh SY, Lee S, Lee JH, Choi HJ. Clinical significance of preoperative neutrophil-lymphocyte versus platelet-lymphocyte ratio in patients with operable colorectal cancer. Biomarkers. 2012; 17 10.3109/1354750x.2012.656705 22424597

[41] Jiang K, Lei J, Li C, Shu K, Li W, Zhang Y, et al Comparison of the prognostic values of selected inflammation based scores in patients with medullary thyroid carcinoma: A pilot study. J Surg Oncol. 2017; 116(3):281–7. 10.1002/jso.24683 28556902

[42] Ying HQ, Deng QW, He BS, Pan YQ, Wang F, Sun HL, et al The prognostic value of preoperative NLR, d-NLR, PLR and LMR for predicting clinical outcome in surgical colorectal cancer patients. Med Oncol. 2014; 31(12):305 10.1007/s12032-014-0305-0 25355641

[43] Song X, Zhang G-M, Ma X-C, Luo L, Li B, Chai D-Y, et al Comparison of preoperative neutrophil–lymphocyte, lymphocyte–monocyte, and platelet–lymphocyte ratios in patients with upper urinary tract urothelial carcinoma undergoing radical nephroureterectomy. OncoTargets and therapy. 2016; 9:1399–407. PMID: PMC4795585 10.2147/OTT.S97520 27042108 PMC4795585

[44] Bhindi B, Hermanns T, Wei Y, Yu J, Richard PO, Wettstein MS, et al Identification of the best complete blood count-based predictors for bladder cancer outcomes in patients undergoing radical cystectomy. Br J Cancer. 2016; 114(2):207–12. 10.1038/bjc.2015.432 26657651 PMC4815810

[45] Yao M, Liu Y, Jin H, Liu X, Lv K, Wei H, et al Prognostic value of preoperative inflammatory markers in Chinese patients with breast cancer. Onco Targets Ther. 2014; 7:1743–52. 10.2147/OTT.S69657 25328407 PMC4196795

[46] Azab B, Shah N, Radbel J, Tan P, Bhatt V, Vonfrolio S, et al Pretreatment neutrophil/lymphocyte ratio is superior to platelet/lymphocyte ratio as a predictor of long-term mortality in breast cancer patients. Med Oncol. 2013; 30(1):432 10.1007/s12032-012-0432-4 23283648

[47] Wariss BR, de Souza Abrahão K, de Aguiar SS, Bergmann A, Thuler LCS. Effectiveness of four inflammatory markers in predicting prognosis in 2374 women with breast cancer. Maturitas. 2017; 101(Supplement C):51–6. 10.1016/j.maturitas.2017.04.015 28539169

[48] Proctor MJ, Morrison DS, Talwar D, Balmer SM, Fletcher CD, O'Reilly DS, et al A comparison of inflammation-based prognostic scores in patients with cancer. A Glasgow Inflammation Outcome Study. Eur J Cancer. 2011; 47(17):2633–41. 10.1016/j.ejca.2011.03.028 21724383

[49] Kou F, Lu Z, Li J, Zhang X, Lu M, Zhou J, et al Pretreatment lymphopenia is an easily detectable predictive and prognostic marker in patients with metastatic esophagus squamous cell carcinoma receiving first-line chemotherapy. Cancer Medicine. 2016; 5(5):778–86. 10.1002/cam4.638 26814381 PMC4864807

[50] Ray-Coquard I, Cropet C, Van Glabbeke M, Sebban C, Le Cesne A, Judson I, et al Lymphopenia as a prognostic factor for overall survival in advanced carcinomas, sarcomas, and lymphomas. Cancer Res. 2009; 69(13):5383–91. 10.1158/0008-5472.CAN-08-3845 19549917 PMC2775079

[51] Goldrath AW, Bevan MJ. Selecting and maintaining a diverse T-cell repertoire. Nature. 1999; 402(6759):255–62. 10.1038/46218 10580495

[52] Hoffmann TK, Dworacki G, Tsukihiro T, Meidenbauer N, Gooding W, Johnson JT, et al Spontaneous apoptosis of circulating T lymphocytes in patients with head and neck cancer and its clinical importance. Clin Cancer Res. 2002; 8(8):2553–62. 12171883

