# Supplementary material for: Prognostic value of systemic inflammatory markers and development of a nomogram in breast cancer
Source: PLoS One. 2018 Jul 26;13(7):e0200936. doi: 10.1371/journal.pone.0200936 (PMC6062056; doi:10.1371/journal.pone.0200936)
Supplement: S1 Fig — (DOCX) [file pone.0200936.s001.docx]

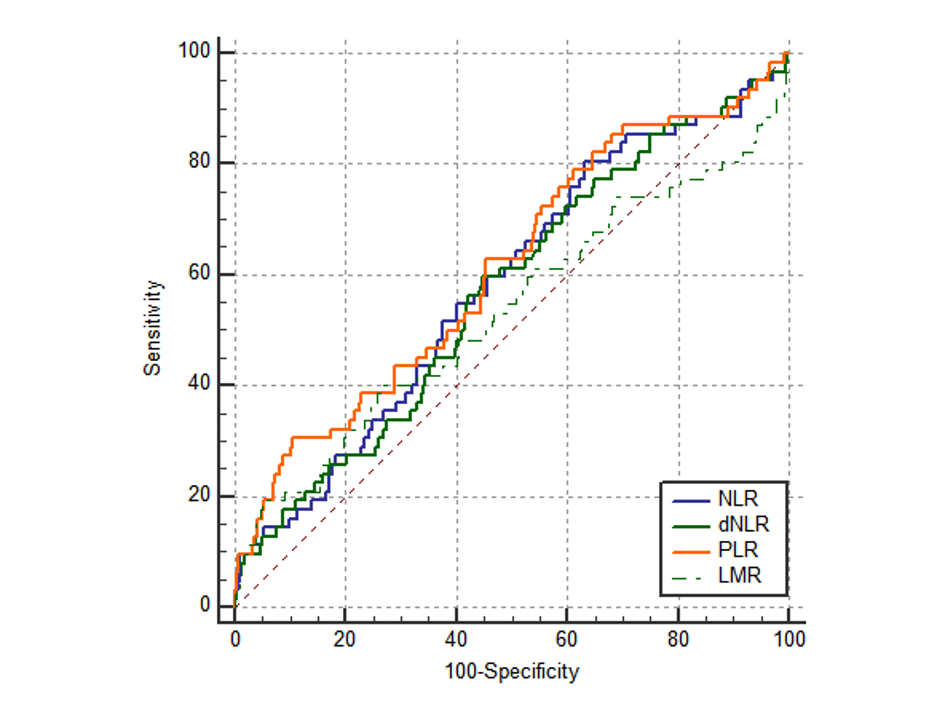


Supporting Fig 1. Receiver operating characteristic (ROC) curves for disease specific survival. The optimal cutoff value was 1.34 for the NLR (sensitivity 80.65, specificity 36.73, AUC 0.58) and dNLR (sensitivity 59.68, specificity 55.26, AUC 0.57), 185.5 for the PLR (sensitivity 30.65, specificity 89.48, AUC 0.61) and 3.11 for the LMR (sensitivity 19.67, specificity 94.99, AUC 0.54).
