# Supplementary material for: Prognostic value of systemic inflammatory markers and development of a nomogram in breast cancer
Source: PLoS One. 2018 Jul 26;13(7):e0200936. doi: 10.1371/journal.pone.0200936 (PMC6062056; doi:10.1371/journal.pone.0200936)
Supplement: S2 Fig — (DOCX) [file pone.0200936.s002.docx]

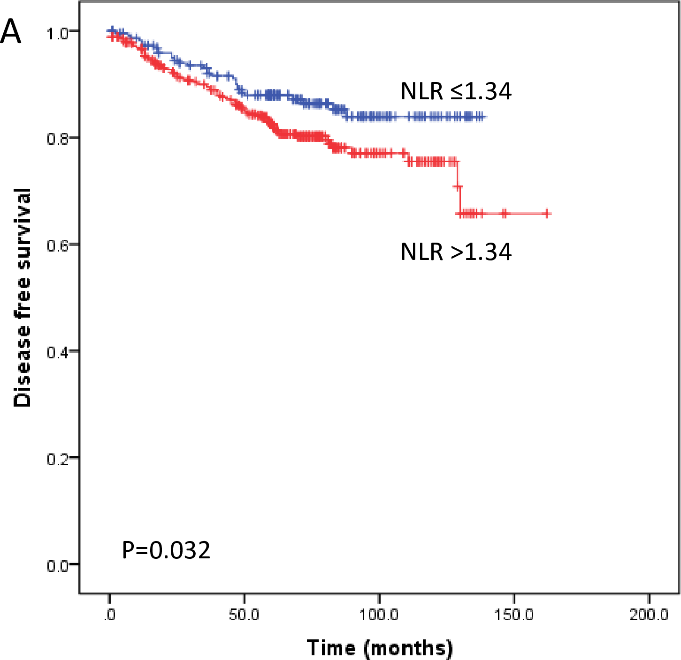

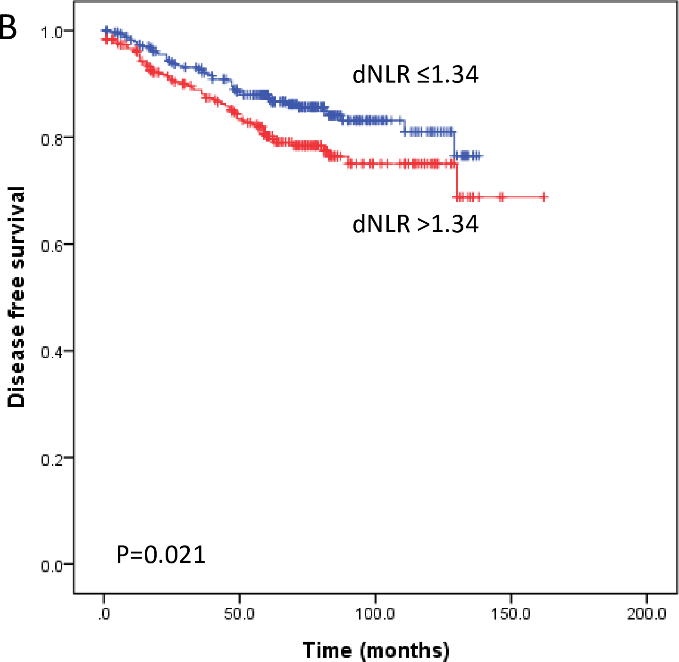

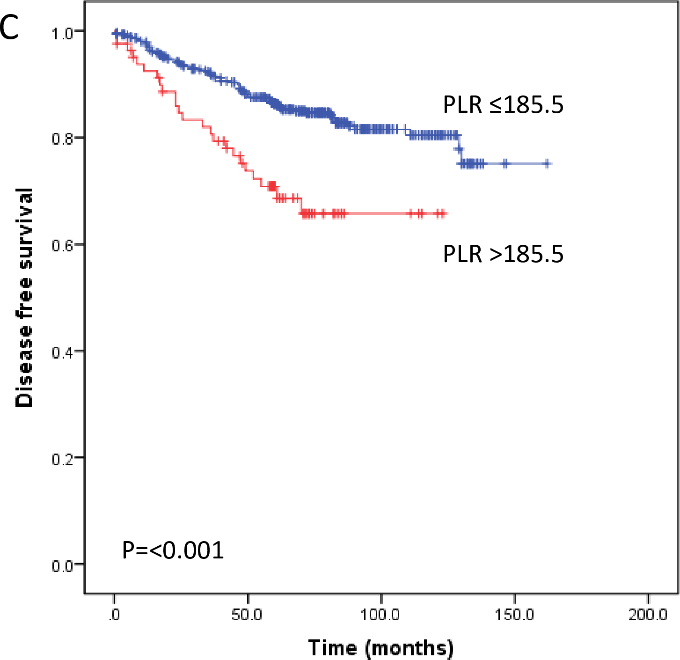

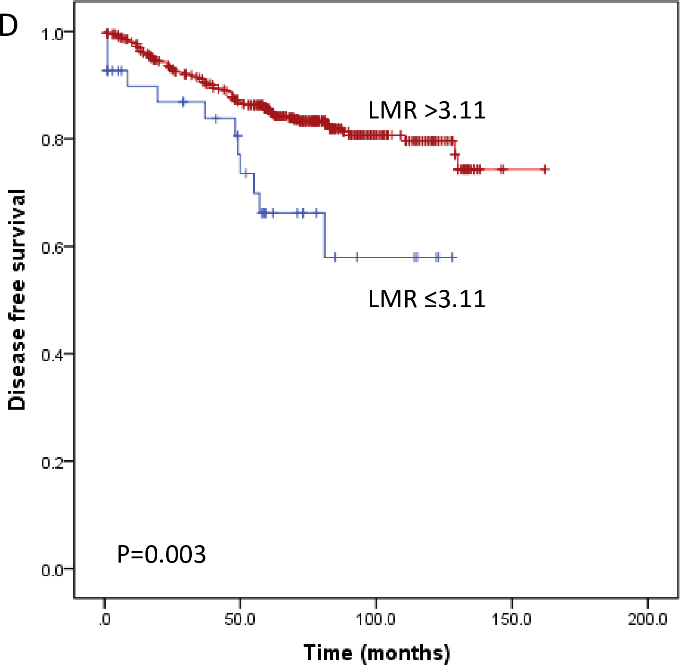


Supporting Fig 2. Kaplan-Meier analyses for disease-free survival of all 661 patients with breast cancer according to the preoperative systemic inflammatory marker. An elevated neutrophil-lymphocyte ratio (NLR) (A), derived neutrophil-lymphocyte ratio (dNLR) (B), and platelet-lymphocyte ratio (PLR) (C) predicted poor disease-free survival following surgical resection. A low lymphocyte-monocyte ratio (LMR) (D) predicted poor disease-free survival.
