# Supplementary material for: Prognostic value of systemic inflammatory markers and development of a nomogram in breast cancer
Source: PLoS One. 2018 Jul 26;13(7):e0200936. doi: 10.1371/journal.pone.0200936 (PMC6062056; doi:10.1371/journal.pone.0200936)
Supplement: S4 Fig — (DOCX) [file pone.0200936.s004.docx]

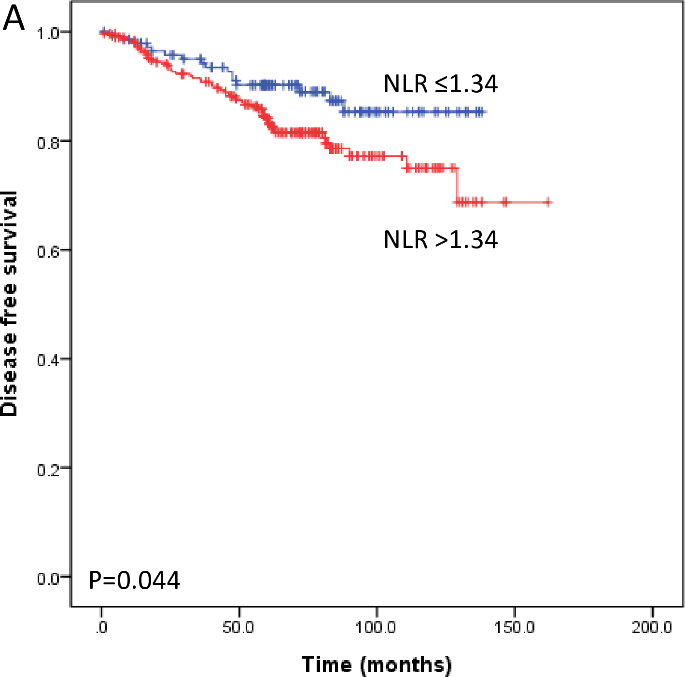

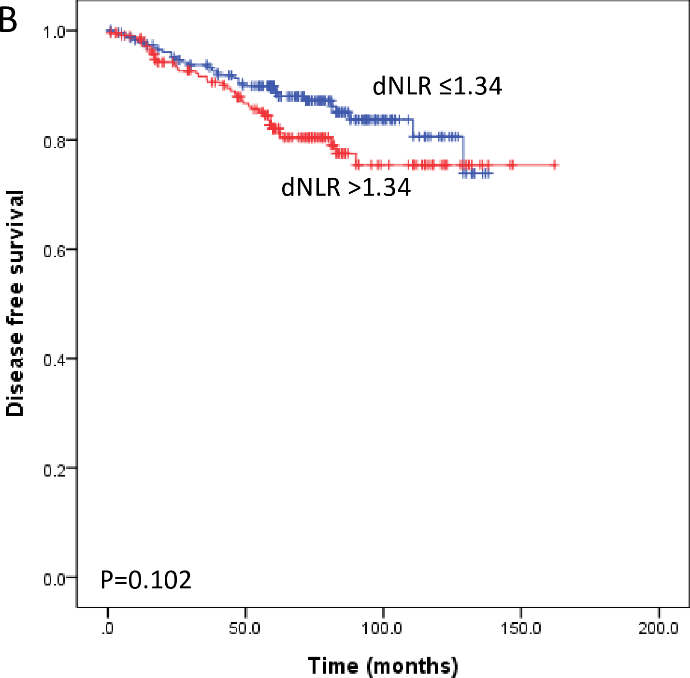

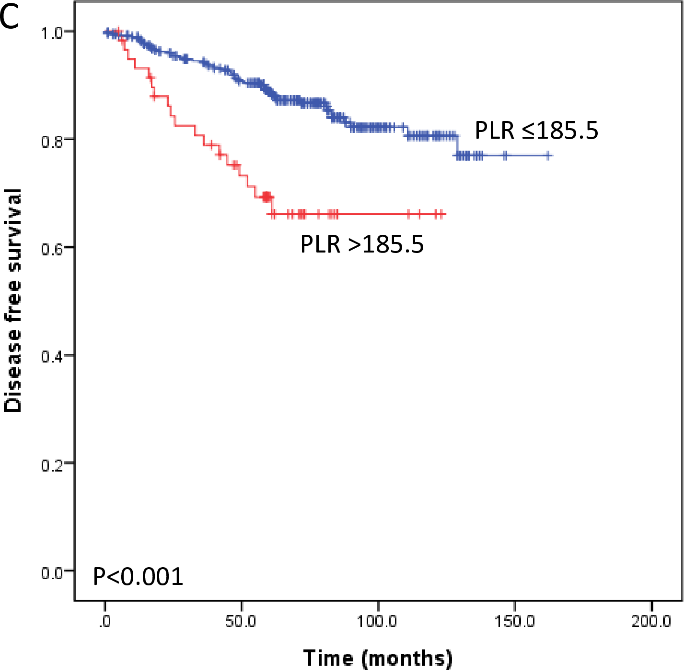

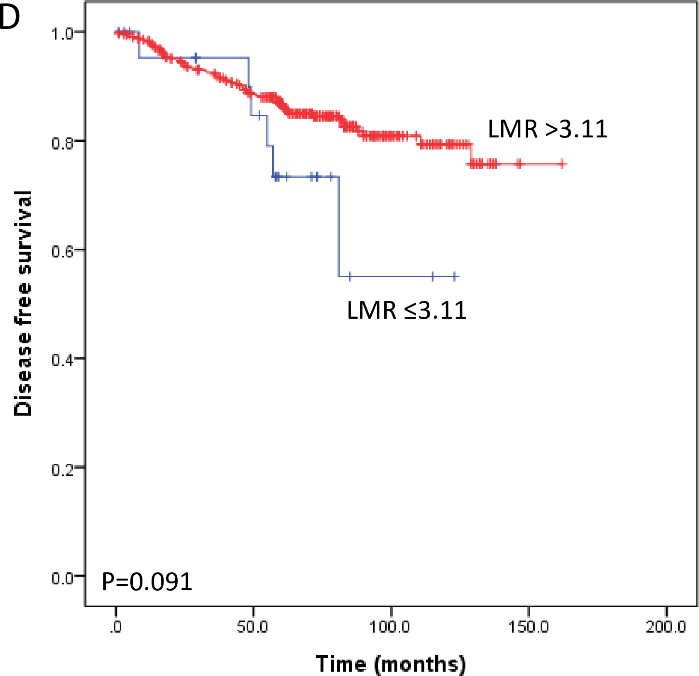
 Supporting Fig 4. Kaplan-Meier analyses for disease-free survival of all 448 patients with luminal type breast cancer, according to the preoperative systemic inflammatory marker. An elevated neutrophil-lymphocyte ratio (NLR) (A) and platelet-lymphocyte ratio (PLR) (C) predicted poor disease-free survival following surgical resection. Whereas, derived neutrophil-lymphocyte ratio (dNLR) (B) and lymphocyte-monocyte ratio (LMR) (D) were not associated with disease-free survival.
